# Endovascular treatment of traumatic azygous vein injuries: a case report

**DOI:** 10.1186/s42155-021-00235-5

**Published:** 2021-06-07

**Authors:** Kristine DeMaio, Shivam Kaushik, Venu Vadlamudi

**Affiliations:** 1 Department of Radiology, University of Tennessee Health Science Center, Methodist University Hospital, 1265 Union Ave, 7 Thomas, Memphis, TN 38104 USA; 2 Rowan School of Osteopathic Medicine, 42 E Laurel Rd, Stratford, NJ 08084 USA; 3 Department of Cardiovascular and Interventional Radiology, Inova Alexandria Hospital, 4320 Seminary Road, Alexandria, VA 22304 USA

**Keywords:** Azygous vein injury, Endovascular management, Endovascular treatment, Endovascular azygous vein injury, Endovascular trauma management

## Abstract

**Background:**

Management of thoracic vascular injury predominantly focuses on the aorta and its tributaries while reports of venous injury are less frequent. Although rare, traumatic azygous vein injuries are associated with high mortality. Prompt treatment is required and has traditionally been open surgery. We present a case of an endovascular repair of an azygous vein injury.

**Case presentation:**

A female patient presented to our trauma center following ejection after a motor vehicle collision (MVC). CT imaging workup revealed mediastinal and periaortic hematoma with active contrast extravasation adjacent to the azygos vein. She was referred to interventional radiology for vascular evaluation and potential endovascular intervention. The patient met criteria for class III hypovolemic shock upon arrival in the endovascular suite. Aortography demonstrated no arterial injury. Venography revealed a pseudoaneurysm on the superior aspect of the azygos arch and contrast extravasation from the inferior margin of the azygous arch. A stent-graft was deployed and post-deployment venogram showed no extravasation and successful exclusion of the injuries. The patient did not have further signs of bleeding. She left the interventional suite with improved vital signs, yet her condition remained guarded. Follow-up CT chest confirmed continued patency of the stent-graft at 8 days and 2 years post-procedure.

**Conclusion:**

Historically, azygos vein injuries are a rare occurrence and managed with open surgery. Swift management is necessary to prevent the increased morbidity and mortality associated with azygous vein injury, particularly in polytrauma patients such as the one presented here. We believe endovascular stent-graft treatment offers an innovative alternative to the current standard of operative management of azygos vein injury.

## Background

Though once thought to be extremely rare, azygous vein injuries have been found in more blunt thoracic trauma patients than previously thought; over an 11-year period at a single institution, seven cases of azygous system venous injury were discovered..(Papadomanolakis et al. 2016) The patients in this report had 100% in-hospital mortality. Of these injuries, only one case had a simultaneous arterial injury – left pulmonary artery laceration (Papadomanolakis et al. 2016). It has been estimated that 45% of thoracic venous injuries die prior to hospital admission, which may be part of why these injuries are seen infrequently (Walsh and Snyder 1992). However, the rate at which these injuries prove fatal, and the additional mortality of open thoracic surgery, underscores the potential benefit of increased use of minimally invasive techniques.

Advances in trauma imaging have made it easier to detect thoracic venous injuries despite limitations in thoracic venous opacification.(Holly and Steenburg 2011) Direct signs of an azygous venous injury include complete tear, rupture, active extravasation, hemothorax, and pseudoaneurysm visualization. Symptoms associated with traumatic venous injury are non-specific, making imaging essential to diagnosis.(Wall et al. 2006)

Previously, surgical approaches were the only described method of treatment despite having a 36% mortality rate.(Wall et al. 2006) We present a case of a successful entirely endovascular repair of a pseudoaneurysm of the superior aspect of the azygous arch and rupture of the inferior aspect of the arch.

## Case presentation

**Fig. 1:**
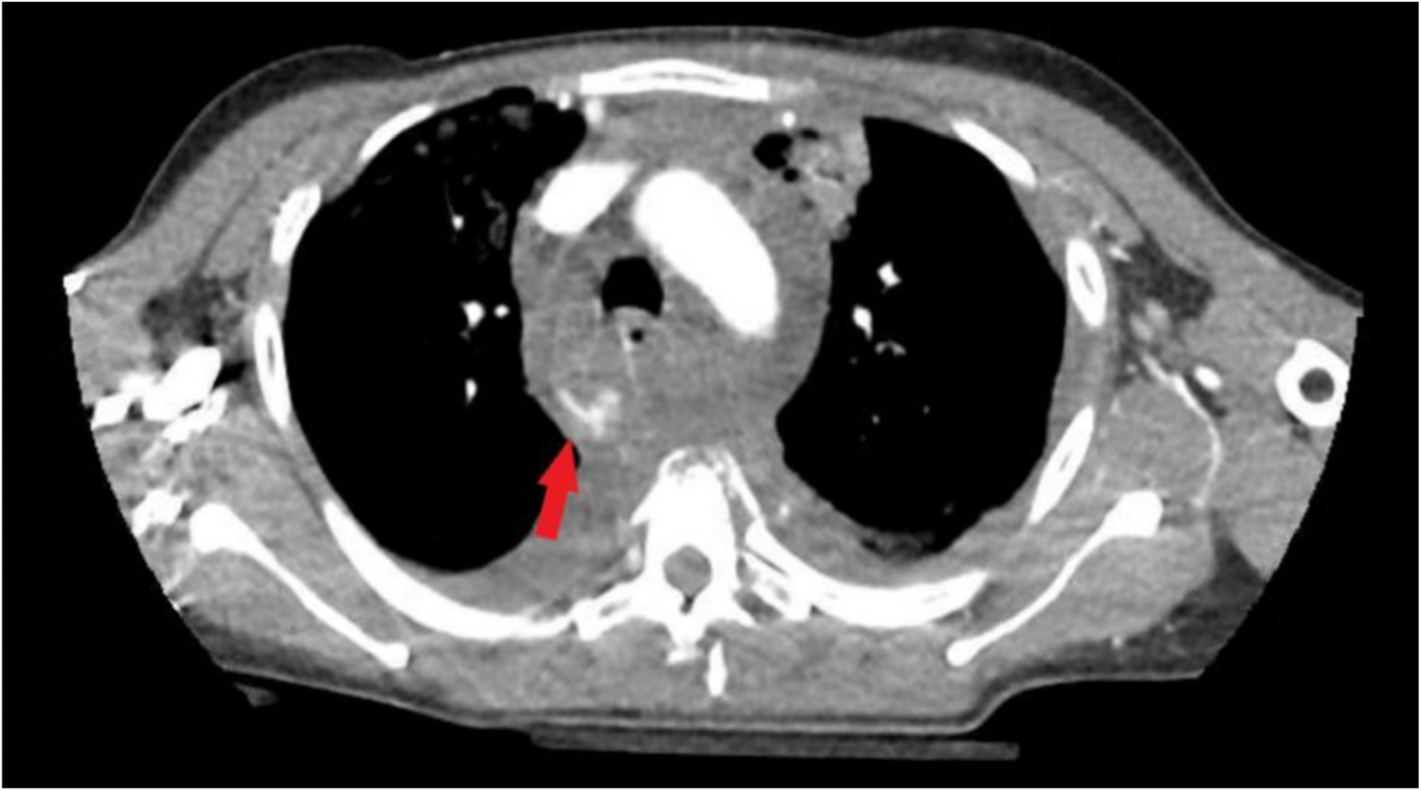
Axial CT slice through the azygous venous arch showing active extravasation of contrast (arrow) from the inferior aspect of the arch

A 28-year-old female presented to our level one trauma center after being ejected 20 yards during an MVC. On arrival she had a Glasgow Coma Score of 14 and was complaining of inability to feel and move her lower extremities, and of back pain worse with movement. She was transported from the trauma bay to the computed tomography (CT) scanner for trauma imaging workup. Her initial CT chest, abdomen, and pelvis with intravenous contrast revealed multiple thoracic injuries including a mediastinal and periaortic hematoma with several areas of contrast blushing, the largest adjacent to the azygos vein suggestive of venous hemorrhage (Fig. 1). Additionally, a sternum fracture, multiple rib fractures, complex T4 and T5 vertebral body fractures with osseous fragment within spinal canal, and concurrent perched facets at this level with multilevel spinous process and transverse process fractures were found. There were trace bilateral hemopneumothoraces and bilateral pulmonary contusions.

Interventional radiology (IR) was then consulted to address the mediastinal hemorrhage concerning for arterial and/or venous injuries. In the interim the patient was transferred to the trauma intensive care unit (TICU) and during this transfer she experienced bradycardia and loss of respirations requiring one round of advanced care life support (ACLS) that resulted in return of spontaneous circulation after intubation. She was also hypotensive to 74/55 mmHg, requiring 5 units of intravenous fluid (IVF) and 3 units of packed red blood cells (pRBC).

Patient arrived in the interventional suite in in class III hypovolemic shock. The right common femoral artery was accessed in standard retrograde fashion. Thoracic aortography was performed in orthogonal left anterior oblique (LAO) and right anterior oblique (RAO) projections. This confirmed normal course and caliber of the thoracic aorta, proximal great vessels, and internal mammary arteries and no extravasation or arterial injury to any of these structures.

**Fig. 2:**
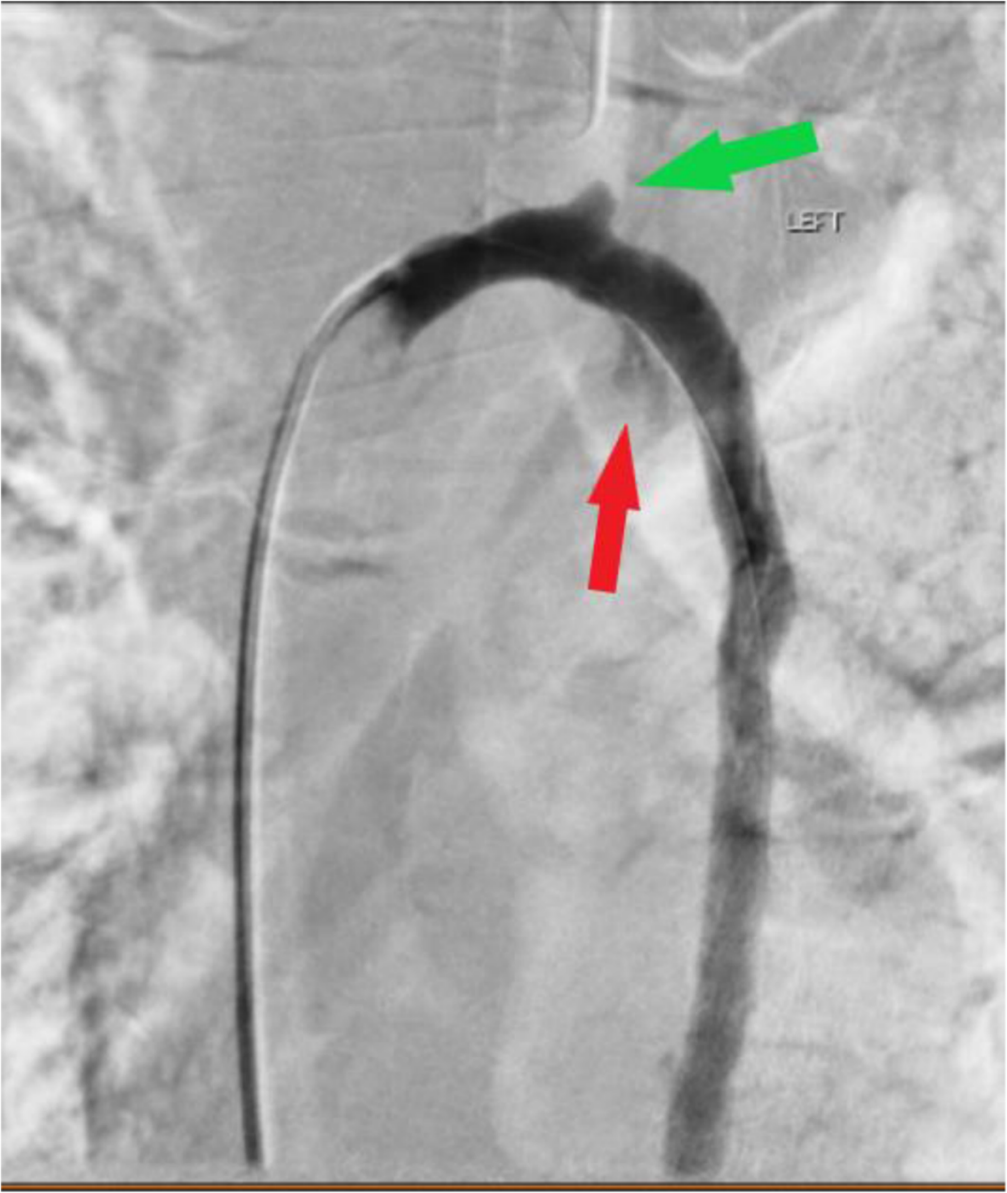
DSA in the LAO projection highlights pseudoaneurysm of the superior arch (green arrow) and extravasation from the inferior arch (red arrow)

After exclusion of a thoracic arterial injury, the right common femoral vein was accessed in standard antegrade fashion. Superior venocavogram was performed in the LAO and RAO orthogonal projections. Due to CT findings suspicious for azygous vein injury, a 5Fr Cobra catheter was introduced over an 035 Glidewire and manipulated across the azygous/superior vena cava (SVC) junction into the distal azygous vein. With an 0.018″ V-18 wire in place, pullback azygous venogram was performed in steep RAO and LAO projections confirming a pseudoaneurysm at the superior aspect of the azygous arch and extravasation from the inferior aspect of the azygous arch (Fig. 2).

**Fig. 3:**
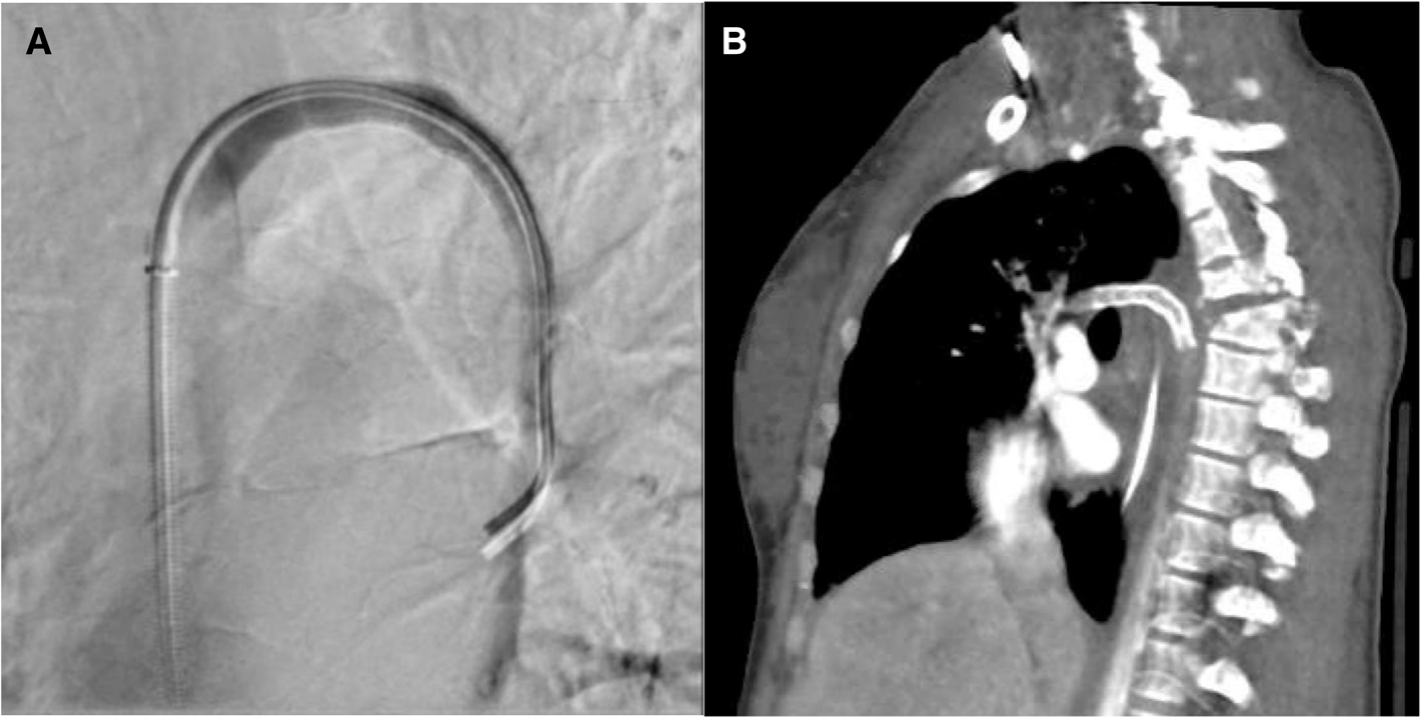
**A** Post procedural DSA confirms no extravasation after Viabahn stent graft placement. **B** CT (sagittal oblique view) performed on post-procedure day eight, though suboptimally timed for venous evaluation, shows no evidence of hematoma, hemorrhage, or other stent malfunction

Based on these injuries, the decision to place a stent-graft across this azygous arch segment was made. The Cobra catheter was advanced back into the azygous vein and the V18 microwire was removed for an exchange length 0.035″ Rosen wire. The Cobra catheter and 5Fr sheath were then removed and an 8Fr × 45 cm Pinnacle Destination sheath was advanced over the Rosen wire and positioned in the mid superior vena cava. Over the Rosen wire, an 8 mm × 5 cm Viabahn stent-graft was positioned across the azygous arch and deployed. Spot fluorography of the chest was obtained, confirming good stent-graft coverage of the areas of injury. Repeat azygous venography with pullback was performed confirming good coverage of the injury sites, no further extravasation, and good antegrade flow of contrast through the azygous arch into the SVC (Fig. 3). The catheter was then removed. The arterial and venous sheaths were removed, and hemostasis was achieved with manual pressure.

The patient tolerated all aspects of the procedure well and there were no immediate complications. She left the procedure suite in guarded condition. The patient had a repeat episode of hypotension after she returned to the TICU, at which time she was started on vasopressors. She was slowly weaned off of all pressors by post-procedure day 6. She underwent several other surgeries and procedures with a variety of services including neurosurgery, general surgery, pulmonology, and insertion of an abdominal drain with IR. She had no complications related to the azygous stent placement which was patent on follow-up CT chest performed on post-procedure day eight. Unfortunately, she remained paraplegic due to her severe spinal injuries.

Additional follow-up was obtained 2 years post-procedure, at which time the stent continued to appear intact and without evidence of endoleak or other malfunction. Patient has had no further medical care or imaging within our system related to her azygous injuries.

## Conclusions

Azygous vein injuries are often fatal, possibly because they are often part of a massive, polytraumatic event.(Papadomanolakis et al. 2016) There are conflicting reports as to whether azygous injuries occur more often in penetrating(Wall et al. 2006) or blunt trauma.(Drac et al. 2007) However, rapid deceleration injury of the azygous vein, as would be experienced in a motor vehicle collision such as the one the patient in this case suffered, occurs due to a well-described combination of three mechanisms: increased venous pressure from compression of the heart between the sternum and spine or compression from the abdominal cavity; secondary injury from fractures of adjacent vertebrae or ribs; and shearing from sudden deceleration since the azygous arch is relatively mobile compared to the heart.(Drac et al. 2007; Haq et al. 2016) Critical clinical signs associated with possible azygous vein injury include shock on arrival to ED, diminished right breath sounds, and hemothorax seen on chest radiograph.(Lyons and Harfouche 2017) Though there are only about 50 published case reports of traumatic azygous vein injury, the 2016 study by Haq et al. performed over an 11-year period found 3.3% of the high-impact torso injuries and 5.0% of the isolated thoracic and thoracic/abdominal traumatic injuries had injuries of the azygous venous system. With the increasing awareness of the azygous vein injury as a cause of morbidity and possibly mortality in the acute thoracic trauma patient, it is likely that these injuries will be detected with increasing frequency.

Thus far only one paper has been published detailing conservative management of an azygous vein injury,(McDermott et al. 2012) while the remainder have undergone open surgical management.(Papadomanolakis et al. 2016) The single case report of successful conservative management of an azygous vein rupture was attempted due to the patient’s hemodynamic stability and CT imaging that demonstrated a non-enlarging azygous hematoma.(McDermott et al. 2012) Patients with hemodynamic instability, high chest tube output, or an enlarging mediastinal hematoma have all been described as needing urgent surgical intervention, almost always a median sternotomy.(Wall et al. 2006; Nguyen and Gates 2006) The endovascular approach used in this case was successful and saved the patient the additional bodily stress and prolonged healing of an open thoracic surgery. Though she did have a protracted, complicated hospital course none of those complications (such as the development of a sacral decubitus ulcer requiring surgical debridement and wound vac placement) were related to the endovascular azygous venous arch repair.

Endovascular techniques, such as described here, should be considered when repairing intrathoracic venous injuries in appropriate cases, such as those in which there would be no other indication for an open thoracic surgery.

## Data Availability

Data sharing not applicable to this article as no datasets were generated or analyzed during the current study.

## Abbreviations

ACLS: Advanced Care Life Support
CT: Computed Tomography
IR: Inteventional Radiology
IVF: Intravenous Fluid
LAO: Left Anterior Oblique
MVC: Motor Vehicle Accident
pRBC: Packed Red Blood Cells
RAO: Right Anterior Oblique
SVC: Superior Vena Cava
TICU: Trauma Intensive Care Unit
